# Assessment of the Impact of a Toll-like Receptor 2 Agonist Synthetic Lipopeptide on Macrophage Susceptibility and Responses to African Swine Fever Virus Infection

**DOI:** 10.3390/v14102212

**Published:** 2022-10-08

**Authors:** Giulia Franzoni, Susanna Zinellu, Elisabetta Razzuoli, Lorena Mura, Chiara G. De Ciucis, Livia De Paolis, Tania Carta, Antonio G. Anfossi, Simon P. Graham, Bernardo Chessa, Silvia Dei Giudici, Annalisa Oggiano

**Affiliations:** 1 Department of Animal Health, Istituto Zooprofilattico Sperimentale della Sardegna, 07100 Sassari, Italy; 2 National Reference Center of Veterinary and Comparative Oncology (CEROVEC), Istituto Zooprofilattico Sperimentale del Piemonte, Liguria e Valle d’Aosta, 16129 Genova, Italy; 3 Department of Biomedical Sciences, School of Medicine, University of Sassari, 07100 Sassari, Italy; 4 Department of Veterinary Medicine, University of Sassari, 07100 Sassari, Italy; 5 Mediterranean Center for Disease Control (MCDC), University of Sassari, 07100 Sassari, Italy; 6 The Pirbright Institute, Ash Road, Pirbright, Woking GU24 0NF, UK

**Keywords:** ASFV, TLR2 agonist, pig macrophages, cytokines, surface markers, pattern recognition receptor

## Abstract

Toll-like receptor 2 (TLR2) ligands are attracting attention as prophylactic and immunopotentiator agents against pathogens, including viruses. We previously reported that a synthetic diacylated lipopeptide (Mag-Pam2Cys_P48) polarized porcine macrophages towards a proinflammatory antimicrobial phenotype. Here, we investigated its role in modulating monocyte-derived macrophage (moMΦ) responses against African swine fever virus (ASFV), the etiological agent of one of the greatest threats to the global pig industry. Two ASFV isolates were compared: the attenuated NH/P68 and the virulent 26544/OG10. No effect on virus infection nor the modulation of surface markers’ expression (MHC I, MHC II DR, CD14, CD16, and CD163) were observed when Mag-Pam2Cys_P48 treated moMΦ were infected using a multiplicity of infection (MOI) of 1. Mag-Pam2Cys_P48 treated moMΦ released higher levels of IL-1α, IL-1β, IL-1Ra, and IL-18 in response to infection with NH/P68 ASFV compared to 26544/OG10-infected and mock-infected controls. Surprisingly, when infected using a MOI of 0.01, the virulent ASFV 26544/OG10 isolate replicated even slightly more efficiently in Mag-Pam2Cys_P48 treated moMΦ. These effects also extended to the treatment of moMΦ with two other lipopeptides: Mag-Pam2Cys_P80 and Mag-Pam2Cys_Mag1000. Our data suggested limited applicability of TLR2 agonists as prophylactic or immunopotentiator agents against virulent ASFV but highlighted the ability of the virulent 26544/OG10 to impair macrophage defenses.

## 1. Introduction

Toll-like receptors (TLRs) are a group of receptors specialized in the recognition of pathogen-associated molecular patterns (PAMPs) that play a crucial role in initiating host immune defenses. TLRs located on the cell membrane (TLR1, -2, -4, -5, -6) recognize microbial lipopeptides or lipopolysaccharides, whereas TLRs localized intracellularly (TLR3, -7, -8, -9) bind nucleic acids [1,2]. Upon PAMP recognition by TLRs, intracellular signaling cascades are initiated, which finally lead to inflammasome activation; the consequent inflammatory responses help limit the progression of invading pathogens [3]. 

TLR agonists are attracting considerable attention as prophylactic and/or therapeutic agents against pathogens, including viruses [4]. Vaccines and antivirals are not available for all viruses, particularly emerging ones, and the evolution and genetic variation of some viruses mean that vaccination alone is not always sufficient for disease control [4,5]. TLR agonists can trigger a rapid activation of the innate immune system, which might be of sufficient intensity to limit pathogen spread within the host [4]. Other studies have investigated the use of TLR-agonists as vaccine adjuvants: these molecules trigger the innate immune defenses, providing a “danger” signal that can improve the development of adaptive immune responses [2].

Several TLR2 agonists have shown promising results against parasites, bacteria, and viruses. For example, macrophage-activating lipopeptide-2 (MALP-2) (a lipopeptide originating from *Mycoplasma fermentans*) showed encouraging immunomodulatory properties in rodents: its intratracheal administration led to recruitment of neutrophils and macrophages into the lung and increased protection against *Streptococcus pneumoniae* [6]. A synthetic analog of this TLR2 agonist, S-[2,3-bis(palmitoyl oxy)propyl] cysteine (Pam2Cys), has been shown to be a potent adjuvant when incorporated into several vaccine candidates [7,8,9]. The pegylated-Pam2Cys (PEG-Pam2Cys) displayed encouraging anti-parasitic activity in mice: it efficiently prevented malaria parasite growth in the liver when administered just six hours before sporozoite challenge [10]. 

In addition, TLR2 ligands showed promising results as prophylactic agents against two zoonotic viruses: SARS-CoV-2 and the influenza A virus (IAV) [11,12,13]. A recent study described that intranasal prophylactic administration of a TLR2/TLR6 agonist (the synthetic diacylated lipopeptide INNA-051) to ferrets restricted SARS-CoV-2 replication in the upper respiratory tract [11]. Furthermore, intranasal administration of PEG-Pam2Cys resulted in the recruitment of innate immune cells in the respiratory tract and promoted the release of proinflammatory cytokines in mice. The PEG-Pam2Cys-induced inflammatory environment protected mice from challenge with IAV [12]. More recently, it was demonstrated that intranasal administration of an engineered Pam2Cys (INNA-X) induced an innate immune response in the upper respiratory tract, which limited IAV infection [5]. In that study, researchers reported that macrophages, in concert with nasal epithelial cells, played a crucial role in limiting virus spread to the lower respiratory tract [5]. 

We previously described that a TLR2 agonist, a chemically synthesized diacylated lipopeptide based on the 14 amino acids following the cysteine immediately downstream of the signal peptide of a surface protein of *M. agalactiae* (P48), polarized porcine macrophages towards a proinflammatory and antimicrobial phenotype [14]. Macrophages are a key element of the innate immune system [15] and are the primary target cells of several viruses, including the African swine fever virus (ASFV) [16,17]. ASFV is the etiological agent of a hemorrhagic disease of suids whose spread has reached pandemic proportions [18]. The disease is currently present in Africa, Europe, and Asia, and outbreaks in the Americas have recently been reported [19]. There are currently no licensed vaccines or treatments available [18], thus the discovery of immunopotentiators, which might be used as prophylactic-metaphylactic agents or vaccine adjuvants, might be able to improve ASFV control, hopefully decreasing the severe associated economic losses. 

Several studies suggested that virulent ASFV isolates have developed mechanisms to escape macrophage immune surveillance, with subsequent abrogation of the development of a protective adaptive immune response, whereas these decoy strategies are partially lost in attenuated strains (such as NH/P68 or OURT88/3) [17,20]. We and others observed that attenuated ASFV strains (OURT88/3 and NH/P68) triggered enhanced expression of cytokines and chemokines (IFNβ, several IFNα subtypes, IL-1β, IL-12p40, TNF-α, CCL4, CXCL8, and CXCL10) compared with virulent ASFV isolates, likely enhancing immune surveillance in vivo, as recently reviewed [17,20]. In addition, we reported that infection with either attenuated (NH/P68) or virulent (22653/14) ASFV impaired the ability of macrophages to secrete pro-inflammatory IL-12, IL-6, and TNF-α in response to stimulation with IFN-γ + LPS or a TLR2 agonist, suggesting that ASFV-infected macrophages were more refractory to external stimuli [21]. Zhu et al. (2019) also observed that virulent Georgia 2007 decreased expression of several TLRs in macrophages, including TLR2, starting from nine hours post-infection (hpi) [22]. 

This study therefore aimed to investigate whether stimulation of macrophages with a TLR2 ligand ‘activated’ these cells against ASFV. The ability of Mag-Pam2Cys_P48 to modulate porcine macrophage responses to ASFV infection was investigated with the aim of investigating its potential use (or that of other TLR2 agonists) as prophylactic or immunopotentiator agents against ASFV.

## 2. Materials and Methods

### 2.1. Animals and Ethical Statement

Seven cross-bred pigs (*Sus scrofa domesticus*) of either sex, aged 6–18 months old, were used as blood donors in this study. Animals were housed at the Experiment Station of the Istituto Zooprofilattico Sperimentale (IZS) of Sardinia (Surigheddu, Sassari, Italy) and their health status was routinely monitored by trained veterinarians. Pigs were screened for ASFV, porcine reproductive and respiratory syndrome virus (PRRSV), porcine parvovirus (PPV), porcine circovirus 2 (PCV2), and *Mycoplasma hyopneumoniae* using commercial real-time PCR kits (LSI VetMAX™ PRRSV EU/NA and VetMAX™-Plus qPCR Master Mix, both from Thermo Fisher Scientific) or qualitative real-time PCR as previously described [23,24], with primers reported in the Supplementary Table S1 [25,26,27]. Animal handling and experimental procedures (bleeding) were approved by the local ethics committee and were authorized by the Ministry of Health (authorization n° 1232/2020-PR). 

### 2.2. Generation of Porcine Monocyte-Derived Macrophages and Polarization

Macrophage cultures were generated from blood monocytes, through the addition of recombinant human M-CSF (hM-CSF) (Thermo Fisher Scientific, Waltham, MA, USA) to culture media, as previously described [14,23,28]. In brief, pig leucocytes purified from heparinized blood were resuspended in RPMI-1640 supplemented with 10% FBS, 100 U/mL penicillin, 100 µg/mL streptomycin (complete RPMI, cRPMI), and hM-CSF (50 ng/mL) and plated in Petri dishes (2 × 10^7^ leukocytes/mL; 20 mL/Petri dish). Cells were incubated for 7 days at 37 °C in 5% CO_2_, then non-adherent leukocytes were removed. Adherent moMΦ were subsequently detached by gentle scraping, centrifuged at 200× *g* for 8 min, re-suspended in cRPMI, and seeded in 12-well plates (Greiner CELLSTAR, Sigma-Aldrich, Saint Louis, MO, USA) (1 × 10^6^ live cells per well) or 4-well chamber slides (Nunc™ Lab-Tek™ Chamber Slide System, Thermo Fisher Scientific) (3 × 10^5^ live cells per well). After plating, cells were incubated for a further 24 h (at 37 °C in 5% CO_2_) before stimulation [14,23].

MoMΦ were left untreated or were treated with a TLR2 agonist S-[2–bis(palmitoyl)-propyl]cysteine (Pam2Cys) lipopeptide: Mag-Pam2Cys_P48 (100 ng/mL). In selected experiments, two other synthetic diacylated lipopeptides were investigated: Mag-Pam2Cys_P80 or Mag-Pam2Cys_Mag1000 (both at 100 ng/mL). These lipopeptides were chemically synthesized based on the 14 amino acids following the cysteine immediately downstream of the signal peptide of three M. agalactiae lipoproteins (P48: CGDKYFKETEVDGV; P80: CVDKDYEELGKDTK; and MAG_1000: CQNDEYQELDYKKW) (Espikem, Prato, Italy) [29]. 

In selected experiments, macrophages were instead classically activated (moM1) using 100 ng/mL of recombinant porcine IFN-γ (Raybiotech Inc, Norcross, GA, USA) and 100 ng/mL of LPS (Lipopolysaccharide from Escherichia coli 0111:B4; Sigma-Aldrich) [21].

### 2.3. Viruses

Two different ASFV isolates were analyzed in this study: the Sardinian field strain 26544/OG10 (GenBank accession number KM102979; ASF Virus Archive, IZS of Sardinia, Sassari) and the low virulence NH/P68 strain (GenBank accession number NC044943; kindly provided by Dr. Carmina Gallardo, EU ASF Reference Laboratory, CISA-INIA, Madrid, Spain). Both strains belong to genotype I, and they were selected based on their differing pathogenicity in vivo: 26544/OG10 induced death in domestic pigs in 10–14 days when administered at a very low dose (10 TCID_50_ by intramuscular injection) (De Mia et al., unpublished results), whereas NH/P68 is characterized by an attenuated phenotype, although it can trigger adverse reactions like fever and joint swelling [30]. For both strains, the working stocks were propagated in vitro on sub-confluent monolayers of two-day-old monocytes/macrophage cultures, using 25 cm^2^ flasks (Corning, New York, NY, USA) [21,23]. Supernatants were collected and pooled with freeze-thawed cell lysates after cells were incubated at 37 °C in 5% CO_2_ for 2–3 days. The obtained pool was clarified from the cellular debris by centrifugation (at 3000× *g* for 15 min) and then divided into aliquots, which were kept at −80 °C until use. Mock virus supernatants were prepared from monocyte/macrophage cultures maintained in the same conditions without viral addition. Titers were determined by the serial dilution of virus suspensions on two-day-old monocyte/macrophage cultures (using 96-well plates, Corning). At 5 days post-infection (pi), virus presence was investigated through both hemadsorption (formation of ‘rosette’) and immunofluorescence staining for 26544/OG10, or immunofluorescence staining for NH/P68 (using a FITC-conjugated anti-ASFV polyclonal antibody, kindly provided by Dr. Feliziani, National Swine Fever Center, CEREP, IZS of Umbria and Marche, Italy), as previously described [23,31]. Viral titers were calculated using the Spearman–Kärber formula [23,31].

### 2.4. Infection of Macrophages

To determine the TLR2 agonists’ impact on moMΦ susceptibility to infection and responses to ASFV strains, moMΦ were seeded in 12-well plates (1 × 10^6^ live cells per well) and left untreated or treated with 100 ng/mL MagPam2Cys_P48 (or MagPam2Cys_P80, MagPam2Cys_MAG1000 in selected experiments). Then, 24 h post-treatment, cells were infected with 26544/OG10 or NH/P68 ASFV at an MOI of 1. After 90 minutes of incubation at 37 °C with 5% CO_2_, the virus inoculum was removed, the cells were washed with RPMI-1640 medium, and fresh cRPMI (1.5 mL/well) was added to the wells. Cells were cultured at 37 °C in 5% CO_2_, and both cells and supernatants were collected at 21 hpi. Cells were stained with different monoclonal antibodies to perform flow cytometry (see Section 2.5), whereas supernatants were collected, clarified from cellular debris through centrifugation at 2000× *g* for 3 min, and then kept at −80 °C until assessment of infectious virus levels by titration (see Section 2.3) and cytokine levels by multiplex or singleplex ELISA (described in Section 2.6) [21,23]. 

To determine TLR2 agonists’ impact on moMΦ ability to support ASFV growth, cells were infected with 26544/OG10 or NH/P68 ASFV at an MOI of 0.01. MoMΦ were cultured at 37 °C in 5% CO_2_, and supernatants were collected at several time points post-infection: 0, 24, 48, and 72 hpi. The supernatants were centrifuged at 2000× *g* for 3 min, divided into aliquots, and stored at −80 °C until the evaluation of infectious virus levels by titration, as described above (see Section 2.3) [21,23].

### 2.5. Flow Cytometry

Flow cytometry was conducted as previously reported [23]. In brief, Zombie Aqua viability dye (BioLegend, San Diego, CA, USA) was used to differentiate live and dead cells. Subsequently, moMΦ were stained with the following murine monoclonal antibodies (mAbs) to quantify surface marker expression: anti-pig MHC I (clone JM1E3, Bio-Rad Antibodies, Kidlington, UK), anti-pig MHC II DR (clone 2E9/13, Bio-Rad Antibodies), anti-human CD14-PerCP-Cy5.5 (clone Tuk4; Miltenyi Biotec, Bergisch Gladbach, Germany), CD16-PE (clone G7, Thermo Scientific Pierce, Rockford, IL, USA), and CD163-PE (clone 2A10/11, Bio-Rad Antibodies). MHC I and MHC II DR expression were subsequently visualized via the subsequent staining with BV421 rat anti-mouse IgG1 (clone A85-1, BD Horizon BD Biosciences, Franklin Lakes, NJ, USA), BV786 rat anti-mouse IgG2b (clone R12-3, BD Horizon BD Biosciences). Details of mAbs are provided in the Supplementary Table S2, and incubations were conducted for 15 min at 4 °C. Surface-stained cells were fixed and permeabilized using Leucoperm (Bio-Rad Antibodies), following the manufacturer’s instructions. Following permeabilization, moMΦ were incubated with anti-p72-FITC (clone 18BG3, Ingenasa, Madrid, Spain) for 30 min to determine intracellular levels of ASFV late viral protein P72. Flow cytometric analyses were carried out using a FACS Celesta (BD Biosciences) and 5000 live moMΦ were analyzed. Analysis of the data was performed using BD FACS Diva Software (BD Biosciences), by the exclusion of doublets, gating on viable moMΦ, and then assessing the staining for surface markers, as we previously described [23]. The gates for late ASFV protein p72 were set using the mock-infected controls; in the ASFV-treated condition, p72 expression was used to discriminate between ASFV-infected (p72^+^) and uninfected bystander (p72^−^) cells [21,23,32]. Gates for surface markers were set using the corresponding unstained/isotype controls, as we previously described [23,32]. 

### 2.6. Cytokine and Chemokine Quantification

Culture supernatants were collected at 24 h post-stimulation and 21 hpi, centrifuged (at 2000× *g* for 3 min), and stored at −80 °C until use. Following the manufacturers’ instructions, [23], the levels of IL-1α, IL-1β, IL-1Ra, IL-6, IL-10, IL-12, IL-18, and TNF-α were determined using the Porcine Cytokine/Chemokine Magnetic Bead Panel Multiplex assay (Merck Millipore, Darmstadt, Germany) and a Bioplex MAGPIX Multiplex Reader (Bio-Rad, Hercules, CA, USA), whereas the levels of IFN-β were assessed using a singleplex ELISA: porcine IFN-β ELISA kit (MyBiosource, San Diego, CA, USA). Levels of chemokines CCL-4 and IP-10 were also evaluated through singleplex ELISA: Porcine CCL-4 or IP-10 ELISA kit (Thermo Fisher Scientific, Waltham, MA, USA), according to the manufacturers’ instructions. For IFN-β, CCL-4, and IP-10, absorbance was read using an Epoch microplate reader (BioTek, Winoosky, VT, USA) [23].

### 2.7. Macrophage Morphology 

MoMΦ were cultured in 4-well chamber slides (3 × 10^5^ live cells per well), and either left untreated or stimulated with TLR2 agonists (Mag-Pam2Cys_P48, Mag-Pam2Cys_P80, Mag-Pam2Cys_Mag1000). To evaluate macrophage morphology 24 h post-stimulation, cells were fixed with 4% paraformaldehyde, washed with PBS, and subsequently phase-contrast images were obtained using an inverted microscope (Olympus IX70, Segrate, Italy) equipped with a 20 X/0.40 numeric aperture objective lens [23]. 

### 2.8. Phagocytosis Assay 

MoMΦ were cultured in 4-well chamber slides (3 × 10^5^ live cells per well), left untreated or stimulated with TLR2 agonists (Mag-Pam2Cys_P48, Mag-Pam2Cys_P80, Mag-Pam2Cys_Mag1000). To evaluate phagocytic ability 24 h post-stimulation, macrophages were incubated with prelabeled red zymosan particles (ab234054, Abcam, Cambridge, UK) for 2 h and then washed by adding cold phagocytosis assay buffer following the manufacturer’s recommendations. Cells were analyzed by fluorescence illumination with an inverted microscope (Olympus IX 70). 

### 2.9. RT-qPCR

Changes in the mRNA expression profiles were monitored as previously described [14]. In brief, moMΦ were seeded in 12-well plates and either left untreated or stimulated with a variety of TLR2 agonists (all at 100 ng/mL); moM1 (IFN-γ/LPS) were also included in the experiment. After 24 h, culture supernatants were removed and cells were lysed using buffer RTL (Qiagen, Hilden, Germany). Then, total RNA was extracted using the RNeasy Mini Kit, treated with the Rnase-Free Dnase Set, and eluted in 50 µL of ultrapure Rnase-free water (all from Qiagen, Milan, Italy). An amount of 250 ng of the obtained purified RNA was used as a template for cDNA synthesis [14]. Then, RT-qPCR was performed to evaluate expression of several genes of the innate immune system (*IL-1β*, *IL-6*, *IL-10*, *IL-12p40*, *TNF-α*, *IFN-β*, *TLR2*, *TLR3*, *TLR7*, *TLR8*, *TLR9*, *cGAS*, *STING*, *RIG-I*, *MDA5*, and *IRF3*), using the primer sets reported in the Supplementary Table S3 [14,33,34,35,36,37]. Real-time PCR amplification was performed in a CFX96™ Real-Time System after the reverse transcription step; glyceraldehyde 3-phosphate dehydrogenase (GAPDH) was used as a housekeeping gene [14]. In each sample, the relative expression of the tested genes was calculated from Cq (quantification cycle) values using the widely adopted 2^−ΔΔCq^ method [14].

### 2.10. Statistical Analysis

In vitro experiments were conducted in technical duplicate and repeated using at least three different blood donor pigs. These data were first checked for normality using the Shapiro–Wilk test and were then graphically and statistically analyzed with GraphPad Prism 8.01 (GraphPad Software Inc., La Jolla, CA, USA). Data were presented as mean and standard deviation (SD) and analyzed using either the parametric one-way ANOVA followed by Dunnett’s multiple comparison test or the non-parametric Kruskal–Wallis test followed by Dunn’s multiple comparison test. 

## 3. Results

We previously described that the diacylated lipopeptide MagPam2Cys_P48 polarized porcine moMΦ toward a pro-inflammatory phenotype [14]. In this work, we investigated whether this synthetic lipopeptide and other TLR2 agonists modulated porcine moMΦ interactions with ASFV.

### 3.1. Impact of MagPam2Cys_P48 on moMΦ Susceptibility to Infection and Responses to ASFV

**Figure 1:**
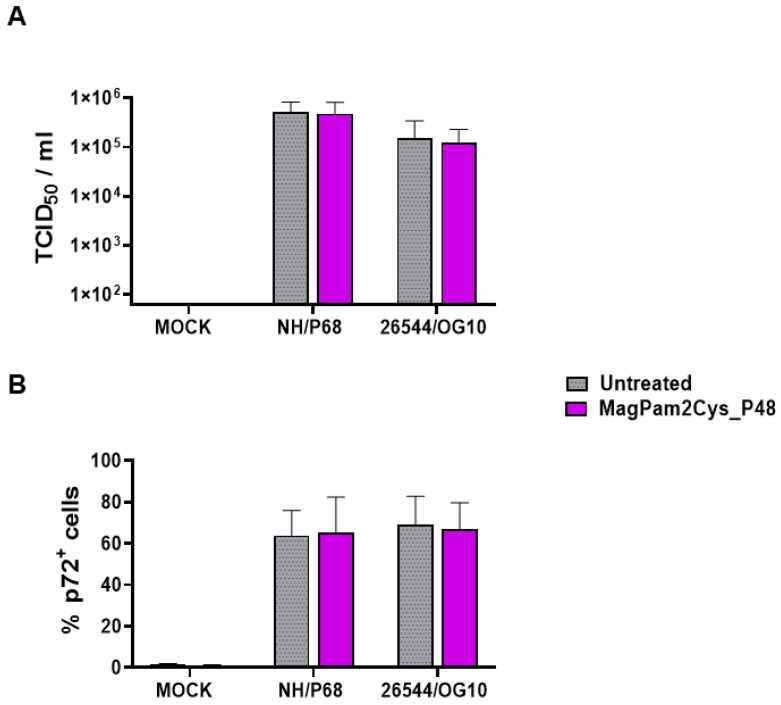
**Impact of MagPam2Cys_P48 on porcine moMΦ susceptibility to ASFV infection.** Porcine moMΦ were left untreated or stimulated with MagPam2Cys_P48 (100 ng/mL). Twenty-four hours later, cells were infected with either attenuated NH/P68 or virulent 26544/OG10, using a MOI of 1. Mock-infected samples were used as controls. At 21 hpi, culture supernatants were collected, and the levels of infectious viral progeny were determined by titration (TCID_50_/mL) (**A**). In parallel, flow cytometry was conducted to determine intracellular levels of ASFV late protein p72 (**B**). The mean data + SD from five independent experiments utilizing diverse blood donor pigs are presented. For each isolate (NH/P68 or 26544/OG10), values of treated macrophages were compared to the corresponding un-treated control (moMΦ), using a Mann–Whitney test or an unpaired *t*-test.

First, the impact of MagPam2Cys_P48 on moMΦ susceptibility to ASFV infection was investigated by quantification of viral titers in cell culture supernatants (Figure 1A) and determination of percentages of cells containing the ASFV late protein p72 (Figure 1B). Cells were left untreated or stimulated with MagPam2Cys_P48. Twenty-four hours later, macrophage subsets were infected with the attenuated NH/P68 or the virulent 26544/OG10 ASFV using a MOI of 1; mock-infected cells were used as a control. At 21 hpi, the amount of infectious virus present in the culture supernatant was determined by titration, whereas flow cytometry was employed to determine the percentages of cells containing the ASFV late protein p72. We observed that no statistically significant differences were detected between TLR2-stimulated or untreated moMΦ for both NH/P68 or 26544/OG10 ASFV (Figure 1).

**Figure 2:**
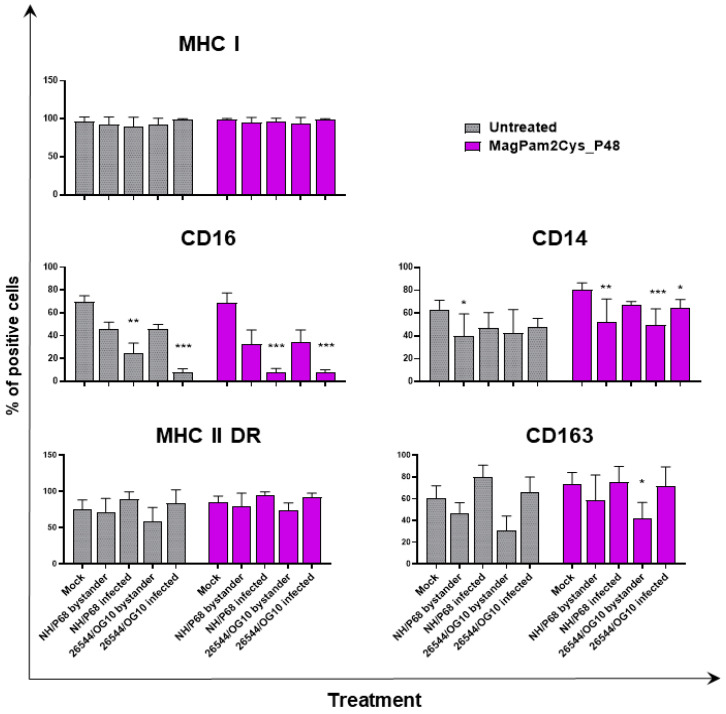
**The effect of MagPam2Cys_P48 on ASFV-modulated expression ofmoMΦ surface markers (percentage of positive cells)**. Porcine moMΦ were left untreated or stimulated with MagPam2Cys_P48 (100 ng/mL). Twenty-four hours later, cells were infected with either attenuated NH/P68 or virulent 26544/OG10, using a MOI of 1. Mock-infected samples were used as controls. At 21 hpi, flow cytometry was employed to determine surface expression of MHC I, CD16, CD14, MHC II DR, and CD163, and intracellular levels of ASFV late protein p72. The mean data + SD from three independent experiments utilizing different blood donors are displayed. For each surface marker, percentages of positive cells are presented. Values of ASFV-infected or bystander cells were compared to the corresponding mock-infected control using a one-way ANOVA followed by Dunnett’s multiple comparison test or a Kruskal–Wallis test followed by Dunn’s multiple comparison test; *** *p* < 0.001, ** *p* < 0.01, * *p* < 0.05.

**Figure 3:**
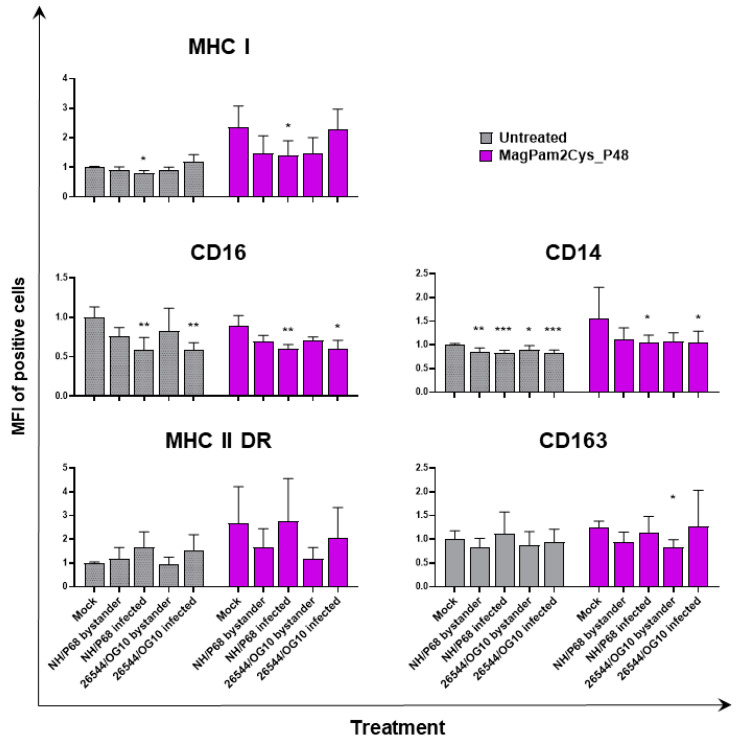
**Impact of MagPam2Cys_P48 on ASFV-modulation of moMΦ surface marker expressions (mean fluorescence intensity).** Porcine moMΦ were left untreated or stimulated with MagPam2Cys_P48 (100 ng/mL). Twenty-four hours later, cells were infected with either attenuated NH/P68 or virulent 26544/OG10, using a MOI of 1. Mock-infected samples were used as controls. At 21 hpi, flow cytometry was employed to determine surface expression of MHC I, CD16, CD14, MHC II DR, and CD163, and intracellular levels of ASFV late protein p72. The mean data + SD from three independent experiments utilizing different blood donors are displayed. For each marker, mean fluorescence intensity (MFI) of positive cells are presented and data are expressed as a fold change relative to the mock-infected un-activated condition (moMΦ mock). Values of ASFV-infected or bystander cells were compared to the corresponding mock-infected control using a one-way ANOVA followed by Dunnett’s multiple comparison test or a Kruskal–Wallis test followed by Dunn’s multiple comparison test; *** *p* < 0.001, ** *p* < 0.01, * *p* < 0.05.

With the aim of further characterizing the effect of MagPam2Cys_P48 on porcine moMΦ responses to ASFV, modulation of the expression of five surface markers and the release of 11 cytokines were analyzed. Macrophage subsets were infected with attenuated NH/P68 or virulent 26544/OG10 using an MOI of 1, alongside mock-infected controls. At 21 hpi, surface expression of MHC class I, MHC class II DR, CD14, CD16, and CD163, and intracellular levels of late viral protein p72 were monitored using flow cytometry, using procedures previously described [21,23]. Using this experimental setup, we observed almost 60% of macrophages expressing ASFV late protein p72 (Figure 1), in accordance with our previous studies [21,23]. Thus, we were able to investigate either the direct effect of ASFV on infected cells (p72^+^) or its impact on bystander uninfected cells(p72^−^) [21,23]. MHC class I and II DR expression was monitored since this can impact antigen presentation. Modulation of CD14 and CD16 expression can impair macrophage anti-microbial/viral activity and previous studies reported that this virus downregulated expression of both markers [17,23]. In addition, ASFV infection can lead to reduced expression of CD163, a scavenger receptor whose expression is regulated by pro- and anti-inflammatory molecules [17,23]. Here we investigated whether MagPam2Cys_P48 alters ASFV modulation of these surface markers. For each marker, both the percentages of positive cells (Figure 2) and the mean fluorescence intensity (MFI) of positive cells were evaluated (Figure 3). Negligible alterations in the expression of MHC class II DR or CD163 on either MagPam2Cys-stimulated or untreated moMΦ were observed following infection with ASFV. In our previous study [21], we described a reduction of CD163 expression 21 h post-infection with 26544/OG10, and in this study we also observed this effect in MagPam2Cys-stimulated moMΦ (Figure 2 and Figure 3). 

In accordance with our previous study [21,23], ASFV infection reduced expression of CD16 on porcine macrophages, and we also observed this effect in MagPam2Cys_P48-stimulated moMΦ. As shown in Figure 2, NH/P68-infected and 26544/OG10 moMΦ pre-stimulated with MagPam2Cys_P48 displayed 7.59 ± 3.76% and 7.61 ± 2.63% of CD16^+^ cells, respectively, drastically lower than that of control (68.78 ± 8.69% CD16^+^ cells). Infection also reduced CD14 expression, observed in either moMΦ (in agreement with [21,23]) or MagPam2Cys_P48-stimulated moMΦ. In the latter subsets, we observed a reduced expression of this marker in either NH/P68-infected (66.75 ± 3.45% of CD14^+^ cells, MFI of CD14^+^ = 1.04 ± 0.16), and 26544/OG10-infected (64.19 ± 7.61% of CD14^+^ cells, MFI of CD14^+^ = 1.04 ± 0.25) moMΦ compared to control cells (80.47 ± 5.96% of CD14^+^ cells, MFI of CD14^+^ = 1.56 ± 0.65) (Figure 2 and Figure 3). In agreement with our previous work [23], we observed that infection with attenuated NH/P68 but not virulent 26544/OG10, triggered MHC class I down-regulation on p72^+^ cells (appreciated in terms of MFI value of MHC I^+^ cells) (Figure 3). As expected, MagPam2Cys_P48 stimulation resulted in enhanced expression (MFI) of this ubiquitously expressed marker on moMΦ (MFI of MHC I^+^ mock-infected cells = 2.35 ± 0.73), infection with attenuated NH/P68 reduced MHC I expression regardless of macrophage activation status (MFI of MHC I^+^ NH/68 infected cells = 1.42 ± 0.48) (Figure 3). 

**Figure 4:**
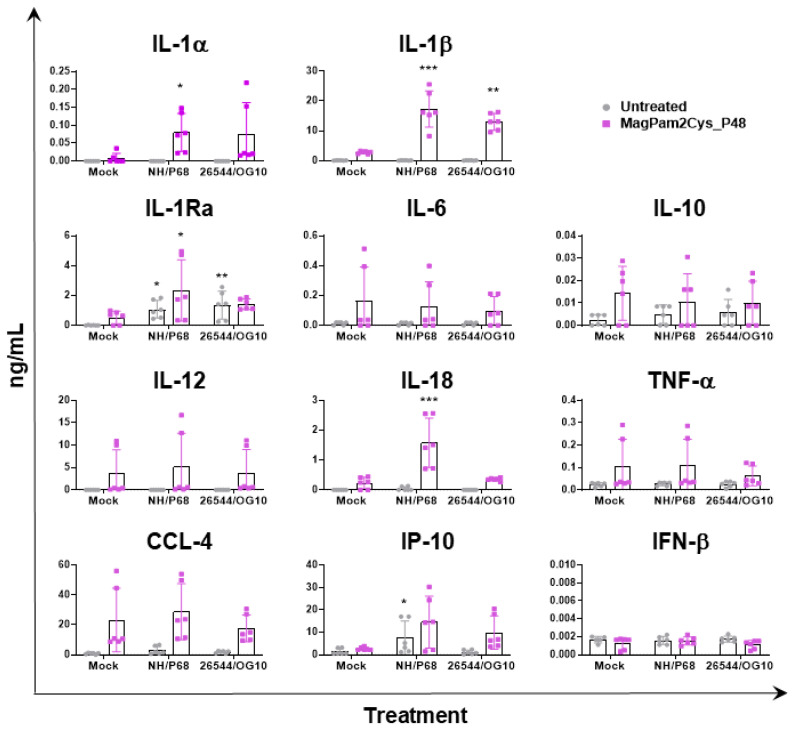
**Impact of MagPam2Cys_P48 on cytokine release by moMΦ in response to ASFV infection.** Porcine moMΦ were left untreated or stimulated with MagPam2Cys_P48 (100 ng/mL). Twenty-four hours later, cells were infected with either attenuated NH/P68 or virulent 26544/OG10, using a MOI of 1. Mock-infected samples were used as controls. At 21 hpi, culture supernatants were collected, and levels of IL-1α, IL-1β, IL-1Ra, IL-6, IL-10, IL-12, IL-18, TNF-α, IP-10, CCL-4, and IFN-β were determined using a multiplex or singleplex ELISA. The mean data ± SD from three independent experiments using different animals are presented. For each macrophage subset, values of ASFV-infected macrophages were compared to the corresponding mock-infected control, using a one-way ANOVA followed by Dunnett’s multiple comparison test or a Kruskal–Wallis test followed by Dunn’s multiple comparison test; *** *p* < 0.001, ** *p* < 0.01, * *p* < 0.05.

In parallel (21 hpi), cytokine content in culture supernatants was assessed by multiplex or singleplex ELISA. We investigated the release of pro-inflammatory (IL-1α, IL-1β, IL-6, IL-12, IL-18, and TNF- α) and anti-inflammatory (IL-1Ra and IL-10) cytokines as well as chemokines (CCL-4 and IP-10) and type I IFN (IFN-β). Release of these mediators might regulate pathological or protective immune responses associated with infection with virulent or attenuated ASFV. In agreement with our previous study [23], our data revealed that most of the tested cytokines were not released by untreated moMΦ in response to ASFV infection, except for IP-10 and IL-1Ra (Figure 4). Infection with attenuated NH/P68 resulted in a higher release of the chemokine IP-10 compared to mock-infected or 26544/OG10-infected moMΦ, as described in our previous study [23]. In this study, IL-1Ra was included in the cytokine panel for the first time, and we observed that both NH/P68 and 26544/OG10 triggered the release of this cytokine by moMΦ (Figure 4). MagPam2Cys_P48 stimulated moMΦ showed enhanced release of IL-1α, IL-1β, IL-1Ra, and IL-18 in response to the attenuated NH/P68 compared to mock-infected cells (Figure 4).

### 3.2. Impact of MagPam2Cys_P48 on moMΦ Ability to Sustain ASFV Replication

**Figure 5:**
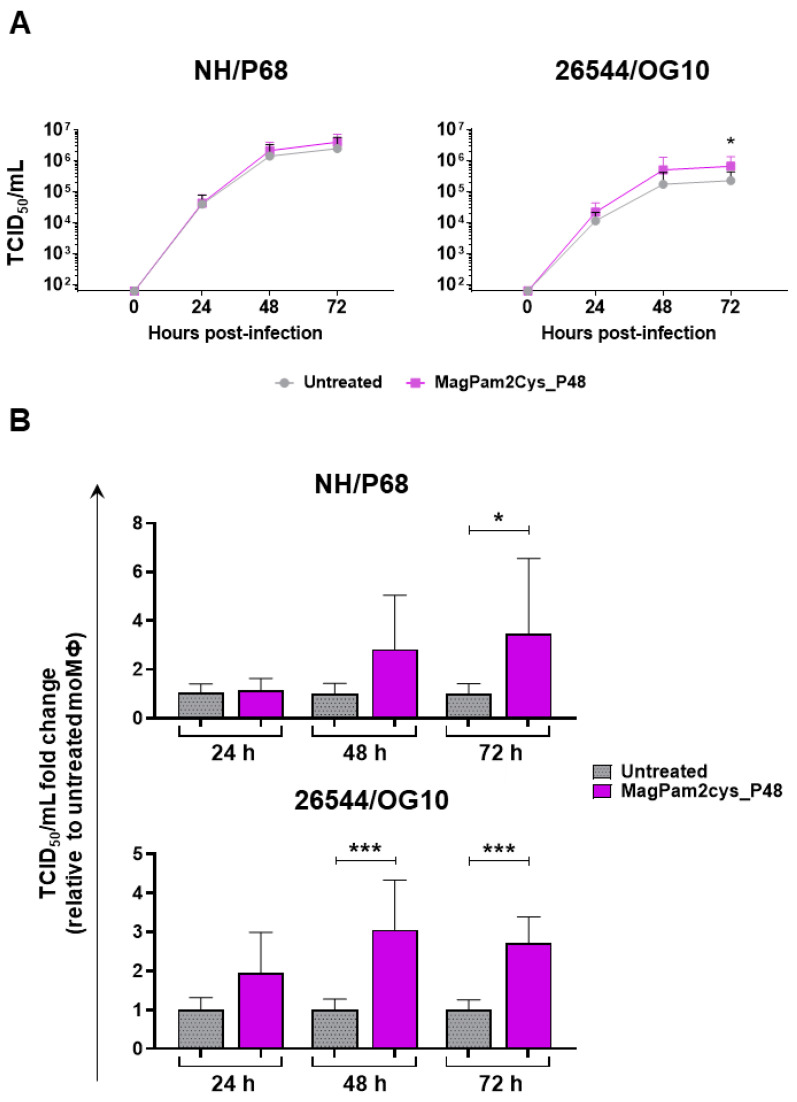
**Effect of MagPam2Cys_P48 on porcine moMΦ ability to sustain ASFV replication.** Porcine moMΦ were left untreated or stimulated with MagPam2Cys_P48 (100 ng/mL). Cells were infected with either attenuated NH/P68 or virulent 26544/OG10, using a MOI of 0.01. At 24, 48, and 72 hpi, culture supernatants were collected, and the levels of infectious viral progeny were determined by titration (TCID_50_/mL). The mean data + SD from four independent experiments utilizing different blood donors are shown. In panel (**A**), data are presented as TCID_50_/mL, whereas in panel (**B**), data for each animal is presented as a fold change to the corresponding untreated control (moMΦ). Fold change to untreated moMΦ was determined for each pig as the ratio between treated (MagPam2Cys_P48) and untreated (moMΦ) cells. At each time point and isolate (NH/P68 or 26544/OG10), values of MagPam2Cys_48-treated macrophages were compared to the corresponding untreated control (moMΦ), using a Mann–Whiney test; *** *p* < 0.001; * *p* < 0.05.

A kinetic analysis of the infection with either NH/P68 or 26544/OG10 ASFV in MagPam2Cys_P48-treated and untreated moMΦ was conducted using an MOI of 0.01. We opted to use a low MOI since at 48 hpi, using a MOI of 1, most ASFV-infected macrophages were detached due to cell death, especially with infection with NH/P68, and at 72 hpi, macrophage culture monolayers were completely detached for both strains, as we previously reported [21]. Both isolates replicated efficiently in all macrophage subsets. However, small but statistically significant differences were observed between macrophage subsets for the virulent 26544/OG10. At 72 hpi, stimulation with MagPam2Cys_P48 resulted in increased levels of ASFV infectious viral particles (expressed in TCID_50_/mL) in the supernatants of 26544/OG10-infected moMΦ, but not NH/P68 infected moMΦ (Figure 5a). Whilst all four different blood donor pig derived moMΦ showed enhanced replication of 26544/OG10 following MagPam2Cys_P48 treatment, this was only observed for NH/P68 in some of the tested pigs. When data from each animal were analyzed as a fold-change relative to the corresponding untreated control (moMΦ), statistically significant differences were also observed for the virulent Sardinian isolate at an earlier timepoint (48 hpi) (Figure 5b). At 72 hpi, levels of ASFV infectious viral particles in 26544/OG10 MagPam2Cys_P48-stimulated moMΦ were almost threefold higher than those in the corresponding untreated moMΦ (Figure 5b).

### 3.3. Comparison of Three TLR2 Agonists on moMΦ Responses and Ability to Sustain ASFV Replication

Overall, these results showed that MagPam2Cys_P48 improved porcine moMΦ ability to release several pro-inflammatory cytokines (IL-1α, IL-1β, and IL-18), as well as IL-1Ra, in response to attenuated NH/P68, while also allowing 26544/OG10 ASFV to replicate more effectively in these cells. Subsequently, we tested whether other TLR2 agonists had similar impacts on porcine moMΦ interaction with ASFV. 

First, we compared the ability of MagPam2Cys_P48 with two other chemically synthesized TLR2 agonists, MagPam2Cys_P80 and MagPam2Cys_MAG1000, to modulate porcine moMΦ phenotype and functionality. Cells were left untreated or stimulated with 100 ng/mL of the corresponding diacylated lipopeptide, then their impact on moMΦ phenotype and functionality was assessed. Twenty-four hours post-stimulation, phase contrast microscopy revealed that none of the tested diacylated lipopeptides altered cell morphology (Figure S1a), in accordance with what we previously appreciated for MagPam2Cys_P48 [14]. In parallel, the modulation of expression of three surface markers was investigated through flow cytometry. All the tested TLR2 agonists strongly upregulated expression of CD14 and MHC class II DR, MHC class I (Figure S1b,c).

The effect of the three TLR2 agonists on moMΦ functionality was also investigated. Twenty-four hours post-stimulation, cytokine levels in culture supernatants were assessed by multiplex ELISA. All the tested diacylated lipopeptides triggered release of pro-inflammatory cytokines: IL-1α, IL-1β, IL-6, IL-12, and TNF-α, whereas no statistically significant differences were observed in the IL-10 levels of un-treated and TLR-2 stimulated-moMΦ (Figure S2a). A phagocytosis assay was also conducted. Cells were left untreated or stimulated with 100 ng/mL of the corresponding diacylated lipopeptide. After 24 h, fluorescent microscopy was employed to investigate the phagocytosis activity of these cells. As displayed in Figure S2b, an increased number of moMΦ associated with red-labelled bioparticles was observed in all the TLR2 agonist-treated conditions compared to moMΦ.

**Figure 6:**
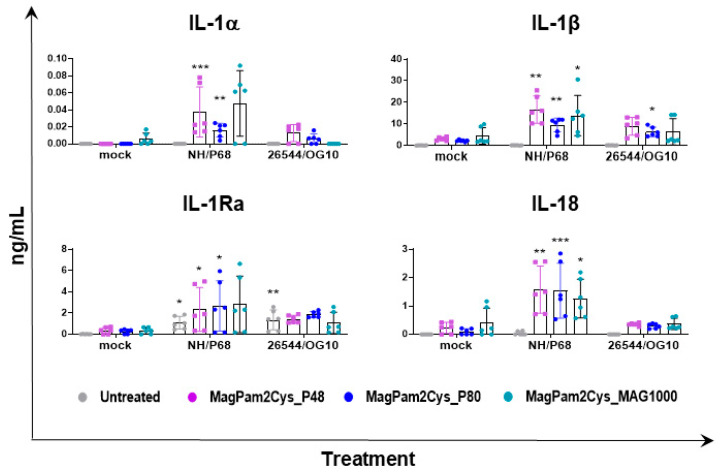
**Effect of MagPam2Cys_P48 on cytokine release by moMΦ in response to ASFV infection.** Porcine moMΦ were either left untreated or stimulated with MagPam2Cys_P48, MagPam2Cys_P80, or MagPam2Cys_MAG1000 (all at 100 ng/mL). Twenty-four hours later, cells were infected with either attenuated NH/P68 or virulent 26544/OG10, using a MOI of 1. Mock-infected samples were used as controls. At 21 hpi, culture supernatants were collected, and levels of IL-1α, IL-1β, IL-1Ra, and IL-18 were determined using a multiplex ELISA. The mean data ± SD from three independent experiments using different animals are presented. For each macrophage subset, values of ASFV-infected macrophages were compared to the corresponding mock-infected control, using a one-way ANOVA followed by Dunnett’s multiple comparison test or a Kruskal–Wallis test followed by Dunn’s multiple comparison test; *** *p* < 0.001, ** *p* < 0.01, * *p* < 0.05.

Overall, our data suggested that all three TLR2 agonists polarized porcine moMΦ in a comparable manner toward a pro-inflammatory antimicrobial phenotype, thus we compared the ability of MagPam2Cys_P48, MagPam2Cys_P80, and MagPam2Cys_MAG1000 to alter moMΦ release of IL-1α, IL-1β, IL-1Ra, and IL-18 in response to ASFV. All three diacylated lipopeptides enhanced the release of pro-inflammatory IL-1α, IL-1β, and IL-18 into the attenuated NH/P68 (Figure 6). Small but statistically significant differences were observed between 26544/OG10-infected and mock-infected moM(MagPam2Cys_P80). As described in Figure 3, both attenuated NH/P68 and highly virulent 26544/OG10 ASFV triggered the release of IL-1Ra. Our data revealed that all tested diacylated lipopeptides increased release of these cytokines in response to NH/P68 infection, whereas no statistically significant differences were observed between 26544/OG10-infected and mock-infected TLR2-treated moMΦ (Figure 6). 

**Figure 7:**
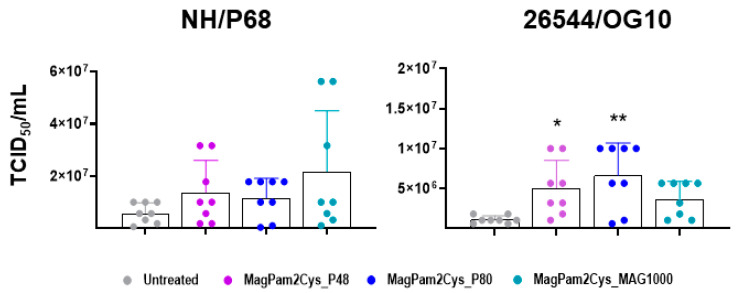
**Impact of diverse synthetic diacylated lipopeptides on porcine moMΦ ability to sustain ASFV replication.** Porcine moMΦ were left untreated or stimulated with MagPam2Cys_P48, MagPam2Cys_P80, or MagPam2Cys_MAG1000 (all at 100 ng/mL). Cells were infected with either attenuated NH/P68 or virulent 26544/OG10, using a MOI of 0.01. At 72 hpi, culture supernatants were collected, and the levels of infectious viral progeny were determined by titration (TCID_50_/mL). The mean data + SD from four independent experiments utilizing different blood donors are shown. For each isolate (NH/P68 or 26544/OG10), values of treated macrophages were compared to the corresponding untreated control (moMΦ), using a Kruskal–Wallis test followed by a Dunn’s multiple comparison test; ** *p* < 0.01, * *p* < 0.05.

Finally, the impact of MagPam2Cys_P48, MagPam2Cys_P80, and MagPam2Cys_MAG1000 on moMΦ ability to sustain ASFV growth was investigated. Treatment of moMΦ with all the tested TLR2 agonists resulted in a small increase in the levels of 26544/OG10 ASFV infectious viral particles in culture supernatants 72 hpi, as displayed in Figure 7 (data expressed as TCID_50_/mL) and Figure S3 (data expressed as fold change relative to the un-activated condition). The enhancement of 26544/OG10 replication was observed in all four tested pigs, whereas our data revealed inter-animal variability for the attenuated NH/P68 (enhancement observed only in three out of four tested subjects). 

### 3.4. Modulation of Key Innate Immune Genes by TLR2 Agonists Potentially Related to Enhanced 26544/OG10 ASFV Replication into Macrophages

Overall, all the tested TLR-2 agonists primed macrophages for enhanced cytokine responses to attenuated NH/P68, similarly to what we previously described for moM1 [21,38]. Surprisingly, stimulation with these molecules resulted in a small, but statistically significant, increase in ASFV 26544/OG10 replication. MoM1 was instead characterized by reduced ASFV replication [21,38], and previous studies reported that TLR2 agonist restricted replication of other viruses [5,11,12], in the final part of the study, we investigated whether modulation of several key genes of the innate immune system by TLR2 agonist was related to the enhanced replication of virulent ASFV. 

Expression of several key cytokines (pro-inflammatory, anti-inflammatory, and type I IFN), TLRs, and other factors critical in the induction of antiviral immunity were investigated by RT-qPCR. MoMΦ were exposed to 100 ng/mL of the corresponding TLR2 agonist; 24 h later, cells were harvested, and RT-qPCR was employed to monitor expression of 15 key immune genes. Untreated cells (moMΦ) and moM1 (stimulated with IFN-γ and LPS) were included in the analysis. 

**Figure 8:**
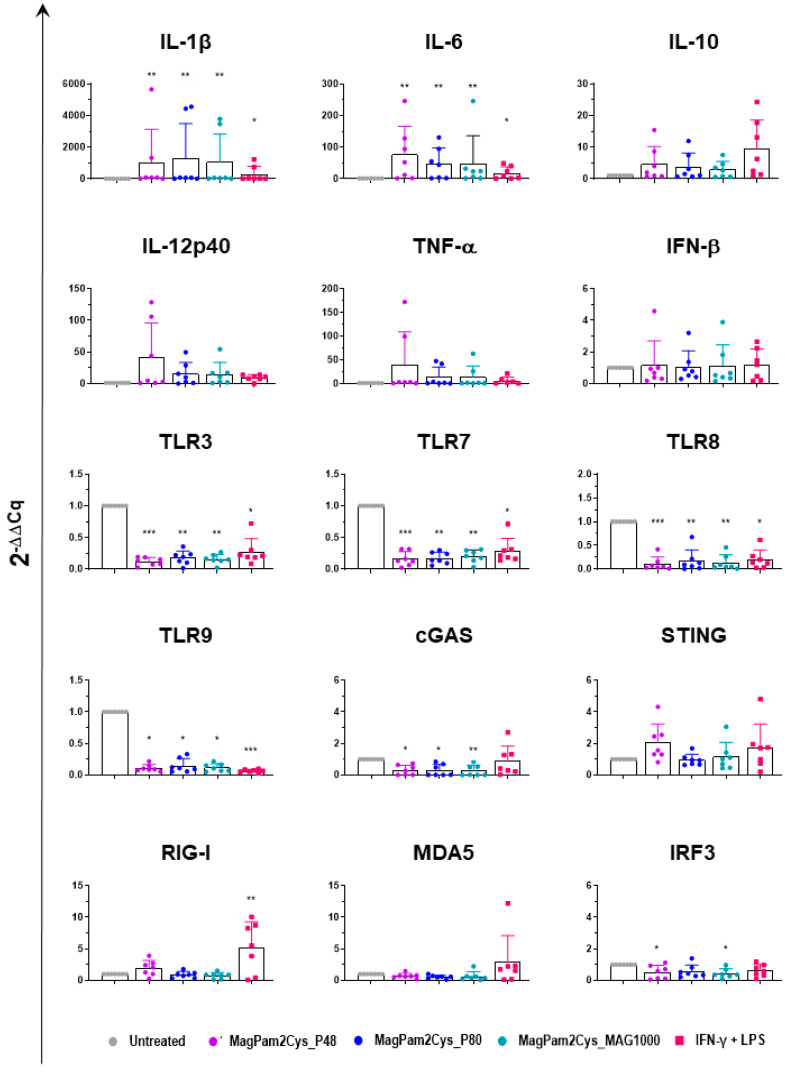
**Modulation of moMΦ innate immune gene expression by diverse diacylated lipopeptides.** Porcine moMΦ were left untreated or stimulated with diverse TLR2 agonists: MagPam2Cys_P48, MagPam2Cys_P80, or MagPam2Cys_MAG1000 (all at 100 ng/mL). MoM1 (generated with IFN-γ + LPS) were included in the experiments. Twenty-four hours later, the expression of *IL-1β*, *IL-6*, *IL-10*, *IL-12p40*, *TNF-α*, *IFN-β*, *TLR3*, *TLR7*, *TLR8*, *TLR9*, *cGAS*, *STING*, *RIG-I*, *MDA5*, and *IRF-3* were determined by RT-qPCR. The data were normalized to the values of the untreated control (moMΦ) and expressed as 2^−^^ΔΔCq^, where ΔCq = Cq (target gene) − Cq (house-keeping gene), and ΔΔCq = ΔCq (stimulated samples) − ΔCq (untreated samples). The mean data + SD from seven independent experiments using different animals are shown. Values of treated macrophages were compared to the untreated control (moMΦ), using a Kruskal–Wallis test followed by Dunn’s multiple comparison test; *** *p* < 0.001, ** *p* < 0.01, * *p* < 0.05.

Our data revealed that all the tested diacylated lipopeptides enhanced gene expression of pro-inflammatory *IL-1β*, *IL-6*, *TNF-α*, and *IL-12p40*, although the latter without statistical significance. A small increase in *IL-10* expression was observed, but it was not statistically significant, and there was no modulation of *IFN-β* expression (Figure 8). Classical activation of macrophages (IFN-γ + LPS) modulates the expression of the six tested cytokines in a similar manner, though it upregulates *IL-1β*, and *IL-6* expression with lower intensity (Figure 8).

In our previous work, we observed that MagPam2cys_P48 downregulated the expression of several TLRs, including intracellular *TLR-3*, *-7*, *-8*, and *-9* [14], and here we compared the impact of this TLR2 agonist to two other diacylated lipopeptides and classical activation. We extended our analyses to TLR2 and other pattern recognition receptors (PRRs). The PRRs involved in pathogen nucleic acid detection were studied, including the RNA sensors RIG-I and MDA5, as well as the DNA sensors cGAS and STING [39]. Modulation of interferon regulatory factor 3 (IRF3), a transcription factor critical in the induction of antiviral immunity, was also investigated [39]. As displayed in Figure 8, we observed that MagPam2Cys_P48, MagPam2Cys_P80, and MagPam2Cys_MAG1000 all significantly downregulated expression of these intracellular TLRs, whereas they did not alter *TLR2* expression (Figure S4). Our results revealed that all the tested TLR2 agonists downregulated expression of *cGAS*, whereas no modulation of *STING*, *RIG-I*, or *MDA5* was observed. All the diacylated lipopeptides under study induced some downregulation of *IRF3*, although with statistical significance only for MagPam2Cys_P48 and MagPam2Cys_MA1000 (Figure 8). Classical activation of macrophages similarly downregulated expression of the four tested TLRs, but it did not modulate *cGAS*, and instead it triggered upregulation of *RIG-I* (Figure 8). 

## 4. Discussion

Macrophages are phagocytic cells at the frontline of defense against pathogens. These cells can quickly and modify their phenotype and functionality in response to external stimuli [40]. The two antithetic extremes of activation states are represented by classically (M1) and alternatively (M2) activated macrophages; the first ones are characterized by increased microbicidal or tumoricidal capacity, whereas the latter are associated with mechanisms of wound repair and immunosuppression [41]. Although macrophage polarization in pigs has not been completely described so far, we and others have observed that classical activation (IFN-γ + LPS) in pigs is characterized by the release of pro-inflammatory cytokines and the upregulation of activation markers [14,42,43]. Macrophages present remarkable plasticity and heterogeneity, and different activator(s), such as IFN-γ, LPS, IFN-β, TLR2 agonists, IL-4, IL-10, and TGF-β, polarize porcine macrophages toward a particular phenotype [34,43,44].

As recently reviewed, macrophages represent the primary target cell for ASFV, which plays a crucial role in the immunopathogenesis of the disease [20]. This large complex virus has evolved numerous strategies to inhibit defenses of its main target cell and replicate undisturbed in them [20]. For example, infection with virulent ASFV does not trigger apoptosis, induction/release of pro-inflammatory cytokines, or modulate expression of MHC class I or II molecules [20]. In addition, we reported that ASFV-infected moMΦ presented lower levels of both CD14 and CD16, with a potential negative impact on these cells’ antimicrobial and antiviral activities [20,38]. In addition, Zhu et al. observed that virulent ASFV Georgia 07 decreased expression of *TLR1*, *TLR2*, *TLR4*, and *TLR6* in macrophages, starting from 9 hpi, with likely impairment of macrophage antimicrobial actions [22]. We recently reported that M2 polarization (using IL-4) did not affect macrophage responses and susceptibility to ASFV infection with both attenuated NH/P68 and virulent 22653/14 isolates. In contrast, we and others described that classical activation (M1) of either porcine alveolar macrophages (PAMs) or moMφ resulted in delayed ASFV replication [21,45]. 

TLR2 agonists are attracting increasing attention as prophylactic and/or therapeutic agents against pathogens, including viruses, because they can trigger a rapid activation of the innate immune system, which might be of sufficient intensity to limit pathogen spread within the host [4]. We previously reported that a synthetic TLR2 agonist (MagPam2Cys_P48) polarized porcine moMΦ towards a pro-inflammatory and microbicidal phenotype [14]. Thus this study aimed to investigate whether stimulation of macrophages with MagPam2Cys_P48 ‘activated’ these cells against the virus, enhancing moMΦ ability to block ASFV replication in the host and promoting development of a protective adaptive immune response against this virus. 

We observed that stimulation of moMΦ with MagPam2Cys_P48 did not alter the impact of ASFV infection on surface expression of five key markers nor the susceptibility to ASFV infection (MOI 1, 21 hpi). On the contrary, our data revealed that two tested ASFV isolates differently modulated the release of several IL-1 family members in macrophages in a pro-inflammatory phenotype. MagPam2Cys_P48-stimulated moMΦ released higher levels of pro-inflammatory IL-1α, IL-1β, and IL-18 in response to attenuated ASFV NH/P68. We observed a similar cytokine pattern for moM1 [21], speculating that this might result in enhanced immune surveillance and the induction of protective immune responses. IL-1β is indeed an important pro-apoptotic factor, and its release might foster apoptosis of uninfected bystander cells, limiting ASFV spread into the host, as we previously thought [17,20,21]. IL-18 is instead a potent IFN-γ inducer, able to activate T cells and NK cells in synergy with IL-12 [46], thus its secretion in response to infection with attenuated NH/P68 might promote the development of ASFV-specific T cell responses, limiting virus spread into the host, as we previously speculated [20]. Higher levels of IL-1β were also observed in 26544/OG10-infected TLR2-agonist stimulated moMΦ compared to the corresponding mock-infected control, although with lower levels compared to attenuated NH/P68. In addition, we observed that both strains triggered the release of another IL-1 family member, IL-1Ra. IL-1Ra is a receptor antagonist and it counteracts the pro-inflammatory action of IL-1α and IL-1β. This cytokine competes for the same receptor of IL-1α and IL-1β (IL-1R), but its binding does not trigger any proinflammatory response [46]. During the inflammatory response, IL-1Ra is released to block further IL-1 activity, preventing development of exacerbated immune responses [46]. Recent in vivo experiments suggested that this cytokine plays a pivotal role in ASFV immune-pathogenic mechanisms [47]. Interestingly, this cytokine was also secreted by untreated moMΦ in response to infection with either NH/P68 or 26544/OG10, the latter with higher intensity, supporting the inhibitory action of ASFV virulent isolates on porcine macrophages, with subsequent weakening of immune vigilance [17,20]. The virulent 26544/OG10 triggered the release of anti-inflammatory IL-1Ra from both untreated and MagPam2Cys_P48-stimulated moMΦ, without concomitant significant release of related pro-inflammatory IL-1α, IL-1β, and IL-18. These data highlighted that the virulent 26544/OG10 developed strategies to strongly impair macrophage defenses, even when these cells were polarized to a pro-inflammatory phenotype. 

Our data revealed that both strains efficiently replicated in MagPam2Cys_P48 treated moMΦ, and interestingly, polarization with this TLR2 agonist also slightly enhanced the ability of ASFV 26544/OG10 to replicate in moMΦ; higher levels of ASFV infectious viral particles were detected in the culture supernatants compared to untreated moMΦ at 72 hpi (MOI 0.01) in all the tested subjects. 

We investigated whether other synthetic TLR2 agonists modulated ASFV-moMΦ interactions in a comparable manner. Analyses were carried out on MagPam2Cys_P80 and MagPam2Cys_MAG1000, which presented immunomodulatory properties on other innate immune cells (ovine neutrophils) similar to MagPam2Cys_P48 [29]. Flow cytometric, ELISA, and fluorescence microscopy data revealed that all three diacylated lipopeptides polarized macrophages toward a pro-inflammatory and antimicrobial phenotype, characterized by upregulation of CD14, MHC class I and MHC class II molecules, release of pro-inflammatory cytokines, and enhanced phagocytic activity. However, we observed that all tested diacylated lipopeptides increased the ability of the virulent ASFV isolate to replicate in these cells.

As stated above, in our previous study, we observed that MoM1 was also characterized for enhanced cytokine responses against ASFV [21]. In detail, our data revealed that higher levels of pro-inflammatory IL-1α, IL-1β, and IL-18 were secreted by NH/P68-infected moM1 compared to the virulent 22653/14 and mock-infected control. Despite the similar patterns of cytokine release, moM1 and TLR2 agonists-stimulated moMΦ differ in ASFV growth kinetics: the former macrophage subsets were characterized by a reduced ability of both attenuated NH/P68 and virulent 22653/14 to replicate at 24 and 48 hpi, with no differences at later time pi (72 hpi) [21], whereas all the tested diacylated lipopeptides slightly enhanced virulent ASFV 26544/OG10 replication efficiency (72 hpi).

Thus, in the final part of the study, we employed RT-qPCR to evaluate whether modulation of major immune cytokines, TLRs, or other factors critical in the initiation of antiviral defenses were triggered by TLR2 agonists and may be related to the enhanced ability of the virulent Sardinian isolate to replicate in macrophages. Our data revealed that all the tested diacylated lipopeptides enhanced the expression of pro-inflammatory cytokines, confirming that these molecules promoted in the development of moMΦ with an antimicrobial phenotype, and no modulation of *IFN-β* gene expression was observed. Classical activation of macrophages similarly modulated expression of the six tested cytokines genes, though it upregulated *IL-1β* and *IL-6* at a lower intensity compared to all of the tested TLR2 agonists. These results are in accordance with our previous work, where we reported that stimulation of moMΦ with IFN-γ and LPS resulted in a lower release of both IL-1β and IL-6 compared to stimulation with MagPam2Cys_P48 [14]. 

We observed that all three synthetic lipopeptides downregulated expression of *TLR-3, -7, -8,* and *-9,* in accordance with what was described in our previous study on MagPam2Cys_P48 [14]. Although this might represent an important protective mechanism in vivo because a tight regulation of the pro-inflammatory process is mandatory to avoid the development of pathological inflammatory responses or even autoimmunity [14,48], it might also impair the ability of macrophages to fight intracellular pathogens, including viruses. In this study, we observed that all three synthetic lipopeptides also drastically downregulated *cGAS* expression. As recently reviewed, PRRs involved in recognition of ASFV include TLR3, which senses viral dsRNA, and cGAS-STING, which senses viral DNA in the cytoplasm [49]. Indeed, ASFV encodes genes that inhibit the actions of both TLR3 and cGAS-STING [49,50,51]. Interestingly, moM1 polarization, which is instead associated with impairment of ASFV’s ability to replicate at early time points [21] and characterized by enhanced release of pro-inflammatory cytokines [14], downregulated *TLR3*, *TLR7*, *TLR8*, and *TLR9*, but it did not modulate *cGAS* expression. Thus, we might speculate that the Mag-Pam2Cys-induced downregulation of this important antiviral defense results in a decreased ability of macrophages to sense ASFV, which should be related to the increased ability of the virus to replicate, although future studies are required to test this hypothesis. As stated above, all diacylated lipopeptides tested increased the ability of both attenuated and virulent ASFV isolates to replicate despite polarizing macrophages toward a pro-inflammatory phenotype. We previously observed that TNF-α (a key pro-inflammatory cytokine) enhanced susceptibility of other myeloid cells (monocyte-derived dendritic cells, moDC) to infection with several virulent, but not attenuated, ASFV isolates [32]. In vivo, infection of domestic pigs with virulent ASFV isolates is often characterized by elevated levels of circulating pro-inflammatory cytokines, such as IL-1β, IL-6, and TNF-α [47,52,53], thus further studies should address whether ASFV has developed mechanisms to enhance replication in a proinflammatory environment in order to better understand the immune-pathogenic mechanisms of ASF. 

## 5. Conclusions

Overall, our data suggest limited applicability of the three tested TLR2 agonists as prophylactic or immunopotentiator agents against ASFV, due to their inability to decrease ASFV replication in macrophages. In addition, we observed differences in responses of macrophages activated with TLR2 agonists to ASFV strains of diverse virulence, with attenuated NH/P68, but not the virulent 26544/OG10, triggering downregulation of MHC I expression and release of IL-1α, IL-β, and IL-18 in macrophages in a pro-inflammatory status. Our data also highlighted the potent ability of the virulent 26544/OG10 to impair macrophage defenses even when these cells were polarized to a pro-inflammatory phenotype. An improved understanding of ASFV-macrophage interactions as well as the identification of factors which promote or counteract the ability of this virus to replicate in its target cells are required to aid the rational design of therapeutic strategies against ASFV.

## Data Availability

The data presented in the study are available on request from the corresponding author.

## Supplementary Materials

The following supporting information can be downloaded at: https://www.mdpi.com/article/10.3390/v14102212/s1, Figure S1. Effect of diverse TLR2 agonists on porcine moMΦ morphology and surface marker expressions; Figure S2. Effect of diverse TLR2 agonists on porcine moMΦ cytokine release and phagocytic activity; Figure S3. Impact of diverse synthetic diacylated lipopeptides on porcine moMΦ ability to sustain ASFV replication; Figure S4. Modulation of *TLR2* expression by diverse diacylated lipopeptides.
