# Supplementary material for: Assessment of the Impact of a Toll-like Receptor 2 Agonist Synthetic Lipopeptide on Macrophage Susceptibility and Responses to African Swine Fever Virus Infection
Source: Viruses. 2022 Oct 8;14(10):2212. doi: 10.3390/v14102212 (PMC9610641; doi:10.3390/v14102212)
Supplement: Supplementary file 1 [file viruses-14-02212-s001.zip › viruses-1918714-supplementary.pdf]

viruses MDPI

Supplementary Table S1. Oligonucleotide Primer and Probe sets for detection of several porcine viruses in pig blood.

Virus	Sequences	Reference / Accession
PCV2	F: 5′-TGGCCCGCAGTATTCTGATT-3′<br>R: 5′-CAGCTGGGACAGCAGTTGAG-3′<br>Probe 5′-FAM-CCAGCAATCAGACCCCGTTGGAATG-BHQ1-3′	[25]
PPV	F: 5′-GAAGACTGGATGATGACAGATCCA-3′<br>R: 5′-TGCTGTTTTTGTTCTTGCTAGAGTAA-3′<br>Probe 5′-VIC-AATGATGGCTCAAACCGGAGGAGA-BHQ1-3′	[26]
ASFV	F: 5′-CTGCTCATGGTATCAATCTTATCG A-3′<br>R: 5′-GATACCACAAGATCRGCCGT-3′<br>Probe 5′-FAM-CCACGGGAGGAATACCAACCCAGTG-TAMRA-3′	[27]

Supplementary Table S2. Antibodies used for flow cytometry.

Antibody	Reactivity	Clone	Isotype	Conjugate	Concentration (mg/mL)	Working dilution*
Primary Antibodies						
MHC I	Pig	JM1E3	Mouse IgG1	-	0.1	1/40
CD14	Human	Tuk4	Mouse IgG2a	Per-CP	ND	1/5
MHC II DR	Pig	2E9/13	Mouse IgG2b	-	0.1	1/25
CD163	Pig	2A10/11	Mouse IgG1	PE	ND	1/4
CD16	Pig	G7	Mouse IgG1	PE	ND	1/4
P72	ASFV late protein P72	18BG3	Mouse IgG2a	FITC	1	1/25
Secondary Antibodies						
Anti-IgG1	Mouse	A85-1	Rat IgG1	BV421	0.2	1/25
Anti-IgG2b	Mouse	R12-3	Rat IgG2a	BV786	0.2	1/25

ND: not determined. * 10 µL of diluted antibody were added to cell pellets (100 µL total).

Supplementary Table S3. Oligonucleotide Primer Sets for Evagreen qRT Real-Time PCR in pig moMΦ.

Gene	Sequences	Reference/Accession
IL-1β	F:5′-AATTCGAGTCTGCCCTGTACCC-3′<br>R:5′-TGGTGAAGTCGGTTATATCTTGGC-3′	[33]
IL-6	F:5′-CAGAGATTTTGCCGAGGATG-3′<br>R:5′-TGGCTACTGCCTTCCCTACC-3′	[33]
IL-10	F:5′-AGCCAGCATTAAGTCTGAGAA-3′<br>R:5′-CCTCTCTTGGAGCTTGCTAA-3′	[34]
IL-12p40	F:5′-TCAGGGACATCATCAAACCA-3′<br>R:5′-GAACACCAAACATCAGGGAAA-3′	[34]
TNF-α	F:5′-TGCCTACTGCACTTCGAGGTTATC-3′<br>R:5′-GTGGGCGACGGGCTTATCTG-3′	[35]
IFN-β	F:5′-AGTTGCCTGGGACTCCTCAA-3′<br>R:5′-CCTCAGGGACCTCGAAGTTCAT-3′	[36]
TLR2	F:5′-CGGCTTCCAAGGATGGAGAAA-3′<br>R:5′-TCCAGAGAGTTGACCTTGCAG-3′	NM213761.1
TLR3	F:5′-TGAAGAACTTGATTTCCTTGGCA-3′<br>R:5′- GGCATGAAAACACCCTGGAG-3′	[14]
TLR7	F:5′-GTGGAAATTGCCCTCGTTGT-3′<br>R:5′-GATGGATCTGTAGGGGAGCA-3′	[14]
TLR8	F: 5′-AAGACAACCAGTTACGTGAAATACC-3′<br>R: 5′-GGGTGTTAAAAGATAATGACAGCAC- 3′	[33]
TLR9	F:5′-AGGACTTCATGCCAAACTGC-3′<br>R:5′-CGAGCAAACATCTCCGACTG-3′	[14]
cGAS	F:5′ -TGGAGTGAAATGTTGCAGGAAAGA-3′<br>R:5′ -GGGTCCTGGGTACAGACGTG-3′	XM_013985148
STING	F:5′-GCCTGCATCCATCCATCCCA-3′<br>R:5′ -GCTGCTCTGGTACCTGGAGTG-3′	NM_001142838
RIG-I	F:5′ -GAATCTGCACGCTTTCGGGG-3′<br>R:5′ -CTGCACCTCATCGTCCCTA-3′	NM_213804.2
MDA5	F:5′ -TGCTGTGAAAGCAATGCAGAATC-3′<br>R:5′ -CGAGACGTCCAGACTTGGCT-3′	NM_001100194.1
IFR3	F:5′ -GGGAAGGAGGCGTGTTCGAC-3′<br>R:5′-ACCAGAGGGTGTAGCGTGGT-3′	NM_213770.1
GAPDH	F:5′-ACCCAGAAGACTGTGGATGG-3′<br>R:5′-ACGCCTGCTTCACCACCTTC-3′	[39]

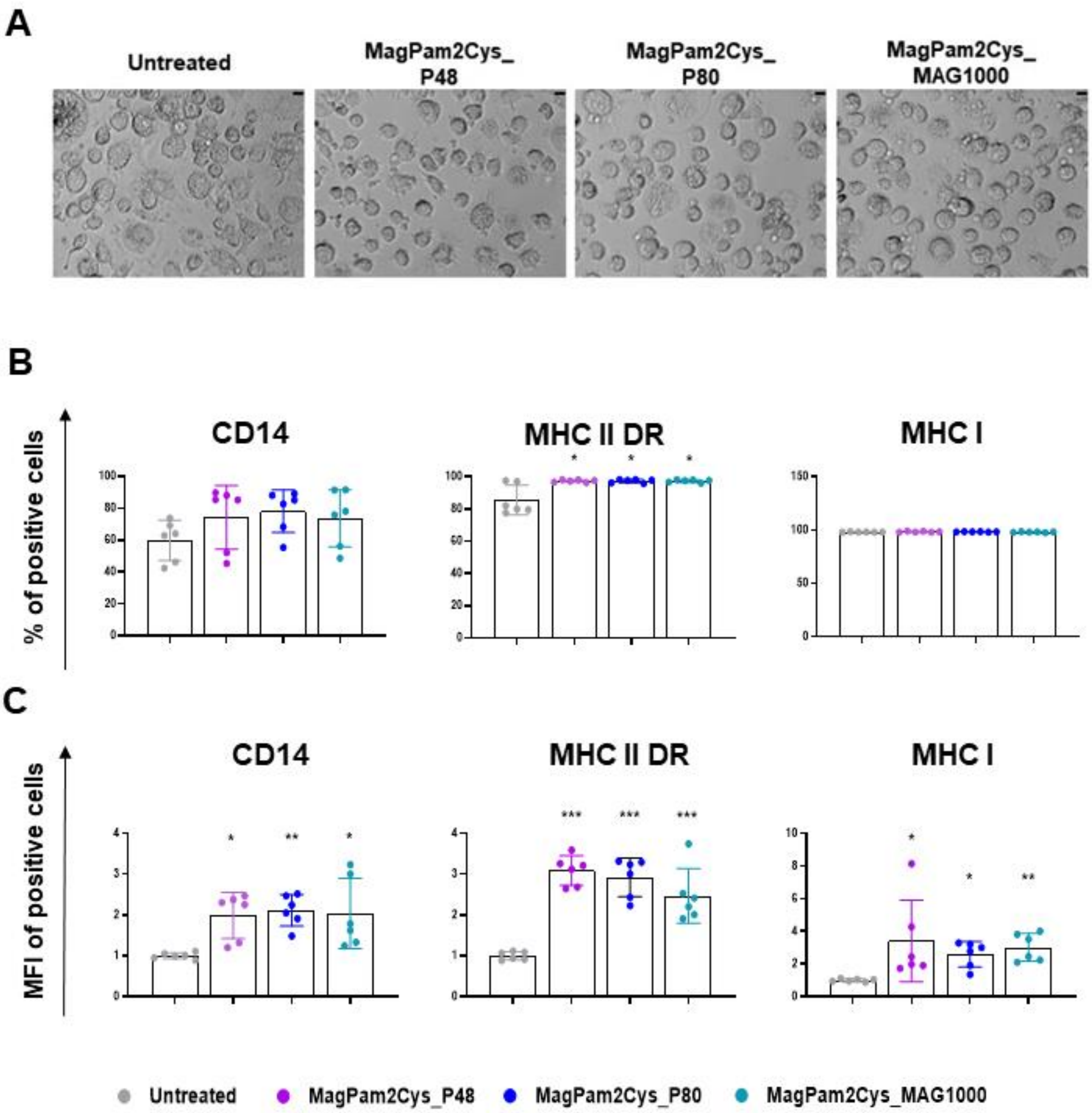

Figure S1. Effect of diverse TLR2 agonists on porcine moMΦ morphology and surface marker expressions. Porcine moMΦ were left untreated or stimulated with 100 ng/mL of diverse TLR2 agonists (MagPam2Cys_P48, MagPam2Cys_P80, MagPam2Cys_MAG1000). (A) 24 h post-stimulation, morphology were evaluated. Phase contrast microscopy images were acquired using an inverted microscope, with a magnification 20x. Scale bar, 10 μm. (B, C) 24h post-stimulation, flow cytometry was employed to determine surface expression of MHC I, MHC II, CD14. For each marker, percentages of positive cells (B) and mean fluorescence intensity (MFI) of positive cells (C) are presented. MFI data are expressed as fold change relative to the mock-infected un-activated condition (moMΦ mock). Values of treated macrophages were compared to the untreated control (moMΦ), using a one-way ANOVA followed by Dunnett's multiple comparison test or a Kruskal-Wallis test followed by Dunn's multiple comparison test; *** $p < 0.001$, ** $p < 0.01$, * $p < 0.05$.

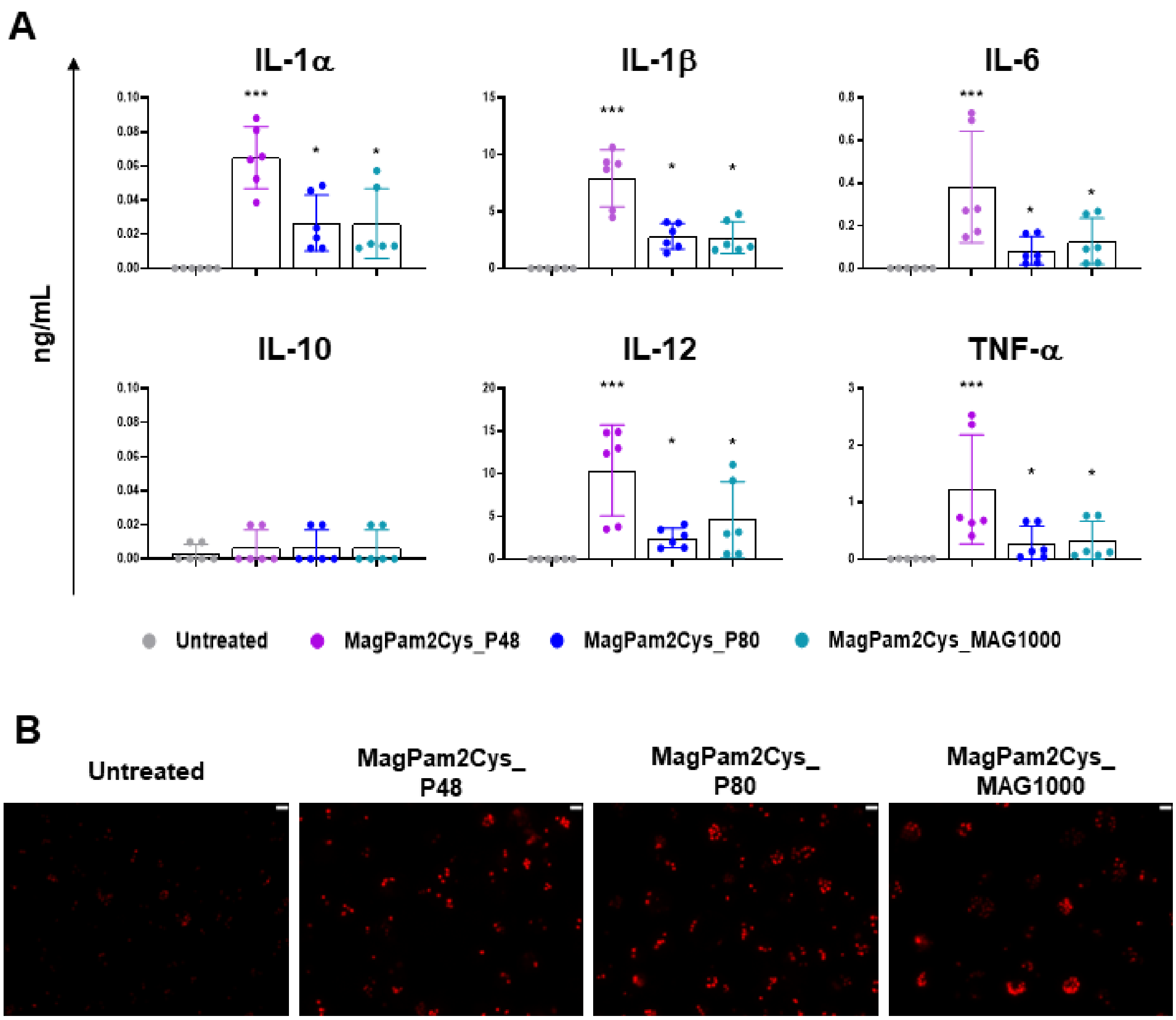

Figure S2. Effect of diverse TLR2 agonists on porcine moMΦ cytokine release and phagocytic activity. Porcine moMΦ were left untreated or stimulated with MagPam2Cys_P48 or MagPam2Cys_P80 or MagPam2Cys_MAG1000 (all at 100 ng/mL). (A) 24h post-stimulation, culture supernatants were collected and levels of IL-1*α*, IL-1β, IL-6, IL-10, IL-12, TNF-*α* were determined using a multi-plex ELISA. For both A and B, the mean data ± SD from three independent experiments utilizing different blood donors are presented. Values of treated macrophages were compared to the untreated control (moMΦ), using a one-way ANOVA followed by Dunnett's multiple comparison test or a Kruskal-Wallis test followed by Dunn's multiple comparison test; *** $p < 0.001$, ** $p < 0.01$, * $p < 0.05$. (B) 24 h post-stimulation, macrophage phagocytotic ability was evaluated using a red zymosan bioparticle conjugated (red). Cells were incubated with bioparticle 2h at 37°C, then images were acquired using a fluorescence microscope, using a 20x magnification. Representative images, one for each condition (untreated, treated with 100 ng/mL of either MagPam2Cys_P48 or MagPam2Cys_P80 or MagPam2Cys_MAG1000) are displayed in panel C. Scale bar 10 μM.

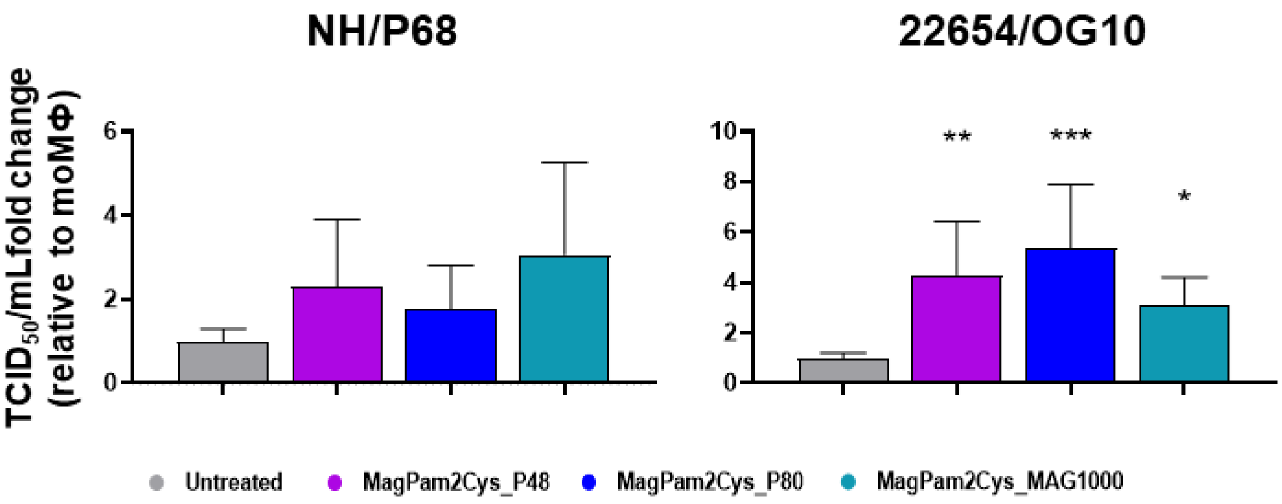

Figure S3. Impact of diverse synthetic diacylated lipopeptides on porcine moMΦ ability to sustain ASFV replication. Porcine moMΦ were left untreated or stimulated with MagPam2Cys_P48 or MagPam2Cys_P80 or MagPam2Cys_MAG1000 (all at 100 ng/mL). Cells were infected with either attenuated NH/P68 or virulent 26544/OG10, using a MOI of 0.01. At 72 h pi, culture supernatants were collected, and the levels of infectious viral progeny were determined by titration ($TCID_{50}$/mL). Data of each animal are presented as fold change to the corresponding untreated control (moMΦ). Fold change to untreated moMΦ was determined for each pig as the ratio between treated and untreated (moMΦ) cells. The mean data + SD from four independent experiments utilizing different blood donors are shown. For each isolate (NH/P68 or 26544/OG10), values of treated macrophages were compared to the corresponding untreated control (moMΦ), Kruskal–Wallis test followed by a Dunn's multiple comparison test; ** $p < 0.01$, * $p < 0.05$.

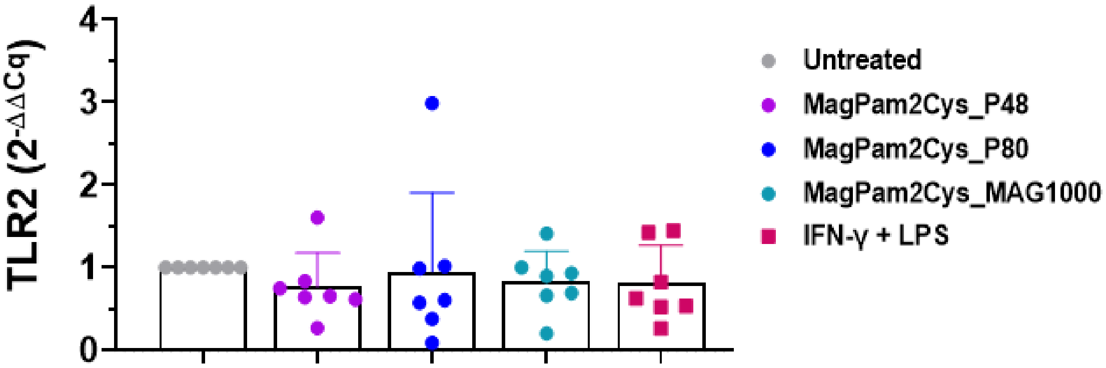

Figure S4. Modulation of TLR2 expression by diverse diacylated lipopeptides. Porcine moMΦ were left untreated or stimulated with diverse TLR2 agonists: MagPam2Cys_P48 or MagPam2Cys_P80 or MagPam2Cys_MAG1000 (all at 100 ng/mL). MoM1 (generated with IFN-γ + LPS) were included in the experiments. 24h later, expression of TLR2 was determined by RT-qPCR. Data were normalized on the values of the untreated control (moMΦ) and expressed as 2-ΔΔCq, with ΔCq = Cq (target gene) − Cq (housekeeping gene), and ΔΔCq = ΔCq (stimulated samples) − ΔCq (untreated samples). The mean data + SD from seven independent experiments using different animals are shown. Values of treated macrophages were compared to the untreated control (moMΦ), using a Kruskal-Wallis test followed by Dunn's multiple comparison test.
